# Sigmoid volvulus and ileo-sigmoid knotting: a five-year experience at a tertiary care hospital in Tanzania

**DOI:** 10.1186/s13017-015-0001-1

**Published:** 2015-03-08

**Authors:** Phillipo L Chalya, Joseph B Mabula

**Affiliations:** Department of Surgery, Catholic University of Health and Allied Science-Bugando, Mwanza, Tanzania

**Keywords:** Sigmoid volvulus, Clinical presentation, Management, Outcome, Tanzania

## Abstract

**Background:**

Sigmoid volvulus is a common cause of intestinal obstruction in developing countries where it affects relatively young people compared to developed countries. No prospective study has been done on this subject in Tanzania and Bugando Medical Centre in particular. This study describes in our region, the clinical presentation, management and outcome of sigmoid volvulus.

**Methods:**

This was a descriptive prospective study of patients operated for sigmoid volvulus at Bugando Medical Centre from March 2009 to February 2014.

**Results:**

A total of 146 patients (M: F = 5.1: 1) representing 14.2% of all cases of bowel obstruction were studied. The median age at presentation was 48 years. The disease significantly affected the older males compared with females (P = 0.012). The majority of the patients 102, (93.2%) presented acutely and had to undergo emergency surgical intervention, the rest were either sub-acute or chronic. Out of the 146 patients studied, 24 (16.4%) had ileo-sigmoid knotting. The majority of patients, 102(69.9%) were treated with resection and primary anastomosis, of which 63.0% were emergency cases. Colostomy was offered to 30.1% of cases. No patient had sigmoidoscopic derotation. Complications mainly surgical site infections were reported in 20.5% of cases. The overall median length of hospital stay was 14 days. Overall mortality rate was 17.1%. The main predictors of mortality were advanced age (>60 years), concomitant medical illness, late presentation (≥24 hours), presence of shock on admission and presence of gangrenous bowel (P < 0.001). The follow up of patients in this study was generally poor as more than half of patients were lost to follow up.

**Conclusion:**

Sigmoid volvulus is not uncommon in our setting and commonly affects males than females. Most of the patients presented acutely, requiring immediate resuscitation and surgical approach. Findings from this study suggest that in viable bowel, sigmoid resection and primary anastomosis is feasible as it may not adversely affect outcome. Temporary colostomy should be considered if the bowel is gangrenous or perforated. Early diagnosis and timely definitive treatment are essential in order to decrease the morbidity and mortality associated with this disease.

## Background

Sigmoid volvulus, first described by von Rokitansky in 1836 [1], is a condition in which the sigmoid colon wraps around itself and its own mesentery, causing a closed-loop obstruction which, if left untreated, often results in life-threatening complications, such as bowel ischemia, gangrene, and perforation [2,3]. It is an important cause of colonic obstruction worldwide [1-3]. In developed countries, sigmoid volvulus ranks the third among large intestine obstructions following cancer and diverticular diseases [4]. It represents 4% of all cases in developed countries and 50% in developing countries [5].

The etiology of sigmoid volvulus is multifactorial and controversial [4,6-9]. Those who possess a sigmoid colon with a long loop and narrow base of mesenteric attachment would be more prone to volvulus [8]. Anatomical predispositions, advanced age, a high-fibre diet, medications altering intestinal motility, chronic constipation, previous abdominal surgery, neurological or psychiatric illness, pregnancy, high altitude and megacolon have all been reported in association with development of the condition [4,6-9].

Sigmoid Volvulus may present with acute sigmoid torsion, recurrent previous torsion or ileosigmoid knotting [10]. Sigmoid volvulus generally affects adults, with the highest incidence seen in the 4th-8th decades of life [5]. However, patients tend to be younger in developing countries as opposed to developed countries where the average age is 62 to 72 years [5,11]. The disease is more common in males and occurs in ratios ranging from 2:1 to 10:1 [1,5,11]. Classically, patients present with a triad of abdominal pain, constipation and abdominal distention [12]. Abdominal X-ray radiograph always revealed findings typical of volvulus in only 65.0% of cases [12,13]. Many other authors have reported similar symptoms and signs plus; vomiting, empty rectal ampulla, associated mental and other medical illnesses in sigmoid volvulus presentation [11-13].

The management of sigmoid volvulus posses a great challenge in resource limited societies as found in Africa [9,13,14]. Late presentation of the disease coupled with lack of advanced diagnostic (such as abdominal Computed Tomography (CT) scan and Magnetic Resonance Imaging ( MRI) and therapeutic facilities for non-operative procedures are a common feature in resource-limited setting like Tanzania [13,14]. Early and correct diagnosis of this disease is essential for appropriate treatment aimed at correcting abnormal pathophysiological changes and restoring intestinal transit caused by the volvulus [2,14].

Despite significant progress in the treatment of this disease, no consensus has been reached [2,14]. Generally, the aim of treatment of sigmoid volvulus is to relieve the obstruction and decompress the twisted sigmoid colon [14]. Many authorities now agree that, in uncomplicated sigmoid volvulus (without perforation or gangrene) sigmoid resection with immediate primary anastomosis is a first choice single-stage operation as it does not increase morbidity or mortality rates [14,15]. On the other hand, if the sigmoid colon is gangrenous then Hartmann’s procedure is recommended [13-16]. Some authors advocate nonoperative such as sigmoidoscopic decompression and derotation as the primary emergency treatment of choice in uncomplicated acute sigmoid volvulus followed by interval semi-elective resection and primary anastomosis several days after successful decompression and emergency surgery is reserved for gangrene or failed decompression [1,17,18]. Emergency surgery is the appropriate treatment for those who present with diffuse peritonitis, intestinal perforation or ischemic necrosis [18,19]. Nonoperative treatment is adopted if there is no evidence of these conditions. However, late presentation of the disease, lack of facilities for nonoperative treatment and associated high rate of recurrence, nonoperative treatment may not be feasible in resource-limited setting.

Sigmoid volvulus is often associated with a high mortality because it affects elderly patients who may have severe co morbid conditions. Patients older than 70 years represent a high risk group if subjected to surgical intervention [19]. However, when volvulus necessitates emergency surgery, it also carries a substantial mortality even in relatively young patients [20]. The highest mortality usually occurs in cases of resection and primary anastomosis of gangrenous sigmoid colon [21].

There is a paucity of information regarding sigmoid volvulus in Tanzania and particularly the study area. This is partly due to a lack of published local data regarding this condition in this region. This study was designed to describe our experience on the management of sigmoid volvulus outlining the clinical presentation, treatment outcome of sigmoid volvulus in our local setting and to identify factors predicting the outcome.

## Methods

### Study design and setting

This was a descriptive prospective study of patients operated for sigmoid volvulus at Bugando Medical Centre from March 2009 to February 2014. Bugando Medical Centre is a tertiary care and teaching hospital for the Catholic University of Health and Allied Sciences-Bugando (CUHAS-Bugando) and has 1000 beds.

### Study population

The study included patients who were operated for sigmoid volvulus at Bugando Medical Centre during the period of study. However, patients aged 10 years and below are usually admitted in the paediatric surgical wards and therefore were excluded from the study. Preoperative diagnosis of sigmoid volvulus was made clinically, radiologically and confirmed at laparotomy. Preoperatively, all the patients recruited into the study were resuscitated with intravenous fluids to correct fluid and electrolyte imbalance; nasogastric suction; urethral catheterization and broad-spectrum antibiotic coverage. Relevant preoperative investigations included packed cell volume, serum electrolytes, urea and creatinine, blood grouping and cross-matching. Radiological investigations including plan abdomen X-ray supine and erect views were done in all patients. Barium enema and Abdominal computed tomography (CT) was done in selected patients. No patients had sigmoidoscopy or Magnetic Resonance Imaging (MRI) investigations due to lack of these facilities’ at our centre.

After resuscitation all patients under general anaesthesia were subjected to exploratory laparotomy through midline incision. They had pre-operative anaesthetic assessment using the American Society of Anaesthetists (ASA) classification [22] as shown in Table 1. To minimize variability in our study, the assignation of ASA class was performed by a consultant anesthetist adhering strictly to criteria above. Adequate hydration was indicated by an hourly urine output of 30 ml/hour. The operations were performed either by a consultant surgeon or a senior resident under the direct supervision of a consultant surgeon.

**Table 1:** **American Society of Anaesthetists (ASA) classification**

**ASA class**	**Description**
I	Healthy individual with no systemic disease
II	Mild systemic disease not limiting activity
III	Severe systemic disease that limits activity but is not incapacitating
IV	Incapacitating systemic disease which is constantly life threatening
V	Moribund, not expected to survive 24 hours with or without operation

Intraoperatively, manual untwisting relieved the obstruction, and the distended hypertrophied sigmoid colon was decompressed by a tube passed through its wall, surrounded by seromuscular purse string of 2/0 vicryl and attached to a suction machine. The contents of the sigmoid colon, primarily gas and liquid feces, were evacuated as much as possible. A nasogastric tube was routinely used in all the cases to decompress the small bowel. The redundant sigmoid colon became evident, and the line of resection was decided. The descending colon and proximal rectum were mobilized, their vascularity was ensured and a resection and two-layered anastomosis with vicryl 2/0 and outer layer of interrupted silk 2/0. If the sigmoid colon was gangrenous, it was resected without untwisting and a Hartmann’s procedure or double barreled colostomy fashioned. The patients underwent 1) emergency or elective sigmoid colon resection and primary anastomosis when the sigmoid colon was viable; 2) sigmoid colon resection and Hartmann’s procedure or double barreled colostomy, when the colon was gangrenous. The peritoneal cavity was lavaged with warm normal saline and the abdomen closed by mass-closure technique. A digital rectal dilatation was carried out as soon as the patient began to recover from anesthesia, to enhance drainage of mucoid colonic contents. Perioperative intravenous antibiotics were given to all the patients in combination with ampicillin 500 mg, gentamicin 80 mg and metronidazole 500 mg. Intravenous ampicillin was given 6 hourly, while gentamicin and metronidazole were given twice or every 8 hours respectively for a period of 72 hours. These were given for a further 48 hours for those with gangrenous bowel. Skin sutures were removed between 7 and 10 days and patients advised on follow-up. Data on each patient were entered into a pro forma prepared for the study. The study variables included socio-demographic (i.e. age and sex, education, area of residence and occupation), associated pre-morbid illness, duration of symptoms, clinical presentation, radiological findings, timing of surgical procedure, ASA classification, operative findings and surgical procedure performed. The variables studied in the postoperative period were postoperative complications, hospital stay and mortality. Patients were followed up till discharge or death and thereafter for a period of six- twelve months.

### Statistical data analysis

Statistical data analysis was done using SPSS software version 17.0 (SPSS, Inc, Chicago, IL). Data was summarized in form of proportions and frequent tables for categorical variables and mode and median for continuous variables. P-values were computed for categorical variables using Chi-square (χ2) test and Fisher’s exact test depending on the size of the data set. Independent student t-test was used for continuous variables. Multivariate logistic regression analysis was used to determine predictor variables that are associated with outcome. A p-value of less than 0.05 was considered to constitute a statistically significant difference.

### Ethical consideration

Ethical approval to conduct the study was obtained from the Catholic University of Health and Allied Sciences/Bugando Medical Centre joint institutional ethic review committee before the commencement of the study. Patients who met the inclusion criteria were requested to sign a written informed consent before being enrolled into the study.

## Results

### Socio-demographic data

During the period of study, a total of 1028 adult patients were admitted to the adult general surgical wards of Bugando Medical Centre and underwent laparotomy for bowel obstruction. Out of these, the underlying cause of obstruction was sigmoid volvulus in 158 patients. Of these, 12 patients were excluded from the study due failure to meet the inclusion criteria. Thus, 146 patients representing 14.2% of all bowel obstruction cases (i.e. 146 out of 1028 patients) were enrolled into the study. The range of patients at presentation ranged from 18 to 82 years with a median age of 48 years (interquartile range, 46 to 52 years). The median age for males (54 years) at presentation was higher than that of their female counterparts (42 years) and this was statistically significant (P = 0.012). The peak age incidence was in the age group 51-60 years. Out of 146 patients, 122 (83.6%) were males and 24(16.4%) were females with a male to female ratio of 5.1: 1. Most of patients, 140 (82.2%) had either primary or no formal education and more than 80% of them were unemployed. The majority of patients, 112(76.7%) came from the rural areas located a considerable distance from the study area and more than three quarter of them had no identifiable health insurance.

### Clinical presentation among patients with sigmoid volvulus

Majority of patients, 136 (93.2%) presented with acute bowel obstruction and the remaining 10 (6.8%) patients presented with sub-acute/chronic bowel obstruction. The duration of symptoms ranged from 1 to 16 days with a median duration of 6 days. Twelve (8.2%) patients presented within twenty-four hours of onset of symptoms, 14 (9.6%) between 24 and 48 hours, 22 (15.1%) between 48 and 72 hours and 98 (67.1%) over 72 hours afterwards. Gross abdominal distention in 140 (95.9%) patients, colicky abdominal pain in 134 (91.8%), constipation in 98 (67.1%), vomiting in 88 (60.3%) and fever in 46 (31.5%) patients were the main symptoms; while dehydration in 68 (46.6%) patients, abdominal tenderness in 60 (41.1%) and visible peristalsis in 62 (42.5%) patients were the main signs. The classic triad of abdominal pain, abdominal distention and constipation was reported in 136 (93.2%) patients. Forty-two (28.8%) of the patients were in shock (with a diastolic blood pressure of less than 90 mmHg) on admission. Twenty-six (17.8%) patients had history suggestive of previous episodes of which eleven (42.3%) had previously been admitted in peripheral hospitals with acute sigmoid volvulus and been managed with laparotomy and de-rotation without resection. Concomitant medical illness such as respiratory diseases (12), cardiovascular diseases (10), diabetes mellitus (8) and renal diseases (5) was reported 35 (24%) patients.

### Diagnosis of sigmoid volvulus

Preoperative diagnosis of sigmoid volvulus was made clinically, radiologically and confirmed at laparotomy. All patients in this study had plain abdominal x-ray films available for review and demonstrated the classical plain abdominal x-ray features of sigmoid volvulus (grossly distended and twisted sigmoid loop filling the abdomen, with multiple air fluid levels and the ‘omega’ or ‘coffee bean’ sign) in 112 (76.7%) patients. Barium enema was done in 12 (8.2%) patients who had no evidence of peritonitis, bowel gangrene, or perforation and demonstrated the obstructive lumen. Abdominal computed tomography (CT) was performed in only 4 (2.7%) patients and demonstrated a twisted and dilated sigmoid colon with whirled sigmoid mesentery, in addition to twisted and dilated small intestinal segments. None of our patients had sigmoidoscopy done due to lack of this facility at our centre.

### Pre-operative anaesthetic assessment

All patients were assessed pre-operatively using the American Society of Anesthetists (ASA) pre-operative grading (Table 1). According to ASA classification, 32 (21.9%) patients had ASA class I, 79 (54.1%) had ASA class II, 24 (16.4%) had ASA class III, 10 (6.8%) had ASA class IV and 1 (0.7%) patients had ASA class V. A high ASA score was found to be an independent predictor of gangrenous bowel (P = 0.000).

### Treatment modalities

All the 146 patients underwent laparotomy. The majority of them, 136 (93.2%) were operated on emergency basis and required immediate resuscitation and relief of the sigmoid obstruction, while 10 (6.8%) patients had an elective surgery. Out of the 146 patients studied, 24 (16.4%) had ileo-sigmoid volvulus. Amongst the patients who had emergency operations, 112 (76.7%) had acute sigmoid volvulus and 24 (16.4%) had ileo-sigmoid volvulus, whereas 10 (6.8%) patients who presented with sub-acute or chronic sigmoid volvulus were operated on elective basis. Table 2 shows distribution of patients according to operative findings. The majority of patients, 102(69.9%) were treated with resection and primary anastomosis, of which 63.0% were emergency cases. Colostomy was offered to 30.1% of cases who had gangrenous and perforated bowel. All the patients who presented with sub-acute obstruction/chronic were treated with primary resection and anastomosis (Table 3). None of our patients in this study had sigmoidoscopic derotation due to lack of this facility at our centre. Delayed presentation (≥24 hours) (P = 0.011) and a high ASA score (P = 0.000) were found to be independent predictors of gangrenous bowel.

**Table 2:** **Distribution of patients according to operative findings**

**Operative findings**	**Frequency**	**Percentages**
**Sigmoid colon (sigmoid volvulus)**	**122**	**83.6**
• Viable	102	69.9
• Gangrenous/perforation	20	13.7
**Ileo –sigmoid portion (ileo-sigmoid knotting)**	**24**	**16.4**
• Viable ileum	1	0.7
• Gangrenous ileum	5	3.4
• Gangrenous both ileum and sigmoid	18	12.3
Peritonitis*	2	1.4
Adhesions*	6	4.1

*Occurred as operative findings in patients with either gangrenous or perforated bowel.

**Table 3:** **Distribution of patients according surgical procedure performed**

**Diagnosis**	**Surgical procedure offered**		**Total**
	**Resection and primary anastomosis**	**Colostomy**	
Acute sigmoid volvulus	92 (63.0)	20 (13.7)	112 (76.7)
Ileo-sigmoid knotting	0	24 (16.4)	24(16.4)
Sub-acute/chronic sigmoid volvulus	10 (6.9)	0	10 (6.9)
Total	102(69.9)	44 (30.1)	146 (100)

### Treatment outcome

A total of 30 (20.5%) patients developed postoperative complications, of which surgical site infection was the most common type accounting for 43.3% (Table 4). Complication rate was significantly higher in emergency operations than in elective operations (32.5% versus 11.9%) (P = 0.014) and in patients with gangrenous bowel undergoing bowel resection (42.3% v/s 13.2%) (P = 0.002). All complications resolved on conservative treatment alone except in three patients who required re-operation for wound dehiscence (2) and intraabdominal abscess (1) respectively.

**Table 4:** **Postoperative complications (N = 30)**

**Postoperative complications**	**Frequency**	**Percentage**
Surgical site infection	13	43.3
Chest infection	4	13.3
Wound dehiscence	2	6.6
Prolonged paralytic ileus	2	6.6
Urinary tract infection	2	6.6
Enterocutaneous fistulae	1	3.3
Intraabdominal abscess	1	3.3

The length of hospital stay (LOS) ranged from 1 to 34 days with a median of 14 days ((interquartile range, 12 to 16 days).The LOS for non-survivors ranged from 1 day to 11 days (median 4 days). The length of hospital stay was significantly longer in patients with advanced age, concomitant medical illness and presence of complications (P < 0.001).

In this study, 25 (17.1%) patients died in the hospital. Amongst the patients treated with primary resection and anastomosis, 17(16.7% i.e 17/102) died while 8(18.2% i.e. 8/44) of those who had colostomy died. This difference was not significant in multivariate logistic regression analysis (P = 0.289). According to multivariate logistic regression analysis, advanced age (>60 years) (OR = 2.5, 95% CI (1.2- 4.8), P = 0.012), concomitant medical illness (OR = 3.2, 95%CI (2.3-5.3), P = 0.003), late presentation (≥24 hours) (OR = 5.4, 95% CI (2.8- 6.9), P = 0.015), presence of shock on admission (OR = 3.2, 95%CI (2.2-8.5), P = 0.001) and presence of gangrenous bowel (OR = 3.2, 95%CI (1.1- 6.8), P = 0.000) were significantly associated with mortality.

### Follow up of patients

Out of the 121 survivors, ninety seven (80.2%) were discharged well, eighteen (14.8%) were discharged home with colostomies and the remaining six (5.0%) patients were discharged against medical advice. No patient among survivors in this study had permanent disabilities. A total of 11 patients had their colostomies closed at the end of study period and the remaining 7 colostomies were not yet closed. The time interval from colostomy creation to colostomy closure ranged from 1 month to 5 months with a median of 4 months (+IQR of 3 to 6 months). Of the 121 survivors, fifty-eight (47.9%) patients were available for follow up at six to twelve months after discharge and the remaining 63 (52.1%) patients were lost to follow up.

## Discussion

Since it was first described by von Rokitansky in 1836 [1], sigmoid volvulus remains a major cause of colonic intestinal obstruction, which results from twisting of the sigmoid colon on its own mesentery [2]. Globally, sigmoid volvulus shows geographic variation being higher in developing countries than in developed world [2,4,7]. It accounts for 2% to 5% of colonic obstructions in Western countries and 20% to 50% of obstructions in Eastern Countries including Africa [4,7]. In this study, sigmoid volvulus accounted for 14.2% of all diagnosed intestinal obstruction seen during the study period in our setting. This concurs with figures of 14.1% that was reported by Jumbi and Kuremu [23] in Kenya. There is no satisfactory explanation for the geographical distribution. It has been suggested that high fiber diet may contribute to the high incidence in Africa where the high fiber results in heavy loading of the sigmoid colon [24,25]. In East Africa, sigmoid volvulus is the second most common cause of intestinal obstruction after adhesions [23].

Sigmoid volvulus has been reported to occur in all age groups, from neonates to elderly [25]. Most often this condition is observed in adults, but the age at which it is most common also varies geographically. In developing countries, a man aged between 40 and 60 years is usually reported, whereas in developed countries, the mean age is between 60 and 70 years [5,11]. As reported in other African studies [11,13,14,23], the median age of 48 years in this study was younger than the age described in most developed countries; about 10 years difference has been reported in these studies [14,23]. We could not establish the reason for this age differences.

The male predominance demonstrated in this study was in keeping with previous observations reported in studies performed elsewhere [11,13,14,19,23]. There is a marked over-all preponderance of male patients with sigmoid volvulus, with a reported ratio of 2.5-9.1 [21,26]. It is suggested that the more spacious female pelvic area allowed a greater possibility of spontaneous reduction of a beginning volvulus [27]. Another predisposing factor is the mesocolon, which is longer in men but wider in women [8]. Heavy loading is more likely to cause sigmoid volvulus in the presence of a longer mesentery.

In keeping with other studies performed in developing countries [14,23], the majority of patients in this series came from the rural areas located a considerable distance from the study area and more than eighty percent had either primary or no formal education. Most of these patients were unemployed and had no identifiable health insurance. This observation has an implication on accessibility to healthcare facilities and awareness of the disease.

Clinically, sigmoid volvulus may present acutely as an emergency or subacutely especially when it is with associated recurrent symptoms of constipation and distention. As reported by other authors in developing countries [13-15], more than ninety percent of patients in this study presented with acute bowel obstruction. In developing countries like ours where over 60% of the populace cannot afford hospital treatment, patients seek hospitalization only when they had developed irreversible intestinal obstruction [13-15]. This observation is reflected in our study where more than ninety percent of patients presented late with acute intestinal obstruction and bowel perforations. The higher incidence rate of delayed presentation in developing countries like Tanzania is best explained by the challenges in health-related transportations, ignorance, poverty and lack of medical awareness [13-15,23]. This delayed presentation increases morbidity and mortality many-folds, as is evident from our results. We could not establish the reasons for the late presentation in this study.

The clinical presentation of sigmoid volvulus in our patients is not different from those in other studies [3-5,11,14,23], with abdominal pain, constipation and abdominal distention being common to all the patients. In this study, the classic triad of abdominal pain, abdominal distention and constipation was reported in 93.2% of the patients.

The diagnosis of acute sigmoid volvulus is established by clinical and radiological findings. In the majority of patients, a thorough physical examination and abdominal radiographs are adequate to achieve the diagnosis. Typical symptoms include sudden abdominal pain and distension followed by constipation. The most common signs are abdominal tenderness and asymmetrical abdominal distention. Other findings include abnormal bowel sounds, abdominal tympany, a palpable abdominal mass, empty rectum, and dehydration [28]. Plain radiographs are diagnostic in 57%-90% of patients [29,30]. The classical sign of acute sigmoid volvulus is the coffee bean sign. Abdominal Computed Tomography (CT) usually reveals a dilated colon with an air/fluid level and the “whirl sign”, which represents twisted colon and mesentery [31]. The classical plain abdominal x-ray features of sigmoid volvulus in this study were demonstrated in more than three quarters of patients. Abdominal CT scan was performed in only 2.7% of cases due to its high cost and irregular availability of this facility at our centre.

Previous studies performed in developing countries showed that ileo-sigmoid knotting accounts for 15-17% of the cases of sigmoid volvulus [11,14,23]. This concurs with our study in which ileo-sigmoid knotting was reported in 16.4% of the patients.

The ileo-sigmoid knot is a rare but serious abdominal emergency in which the ileum and sigmoid entangle each other to form a knot, which may lead to vascular compromise and gangrene of both the ileum and sigmoid colon. The condition is serious, generally progressing rapidly to gangrene of both ileum and sigmoid colon. However, the accurate preoperative diagnosis of ileo-sigmoid knotting is difficult, particularly when abdominal CT is not used. The disease is generally misdiagnosed as an obstructive or non-obstructive emergency in the preoperative period and the correct diagnosis is made upon laparotomy or, in some cases, autopsy [14,19,32]. This observation is reflected in our study in which all patients with ileo-sigmoid knotting presented acutely and all except one were found to have gangrenous bowel. Therefore, awareness of the condition is essential, for prompt diagnosis and optimal management.

The treatment of sigmoid volvulus remains controversial, and depends on the selected procedure and the most appropriate therapeutic approach in terms of the clinical status of the patient, the location of the problem, the suspicion or presence of peritonitis, bowel viability and the experience of the surgical team [33].

Initial nonoperative management, that is, sigmoidoscopic decompression as advocated by Bruudsgaard [34], followed by semi-elective sigmoidectomy and primary anastomosis has been widely accepted as standard management [11,35]. The nonresectional procedures such as sigmoidopexy and mesosigmoidoplasty have no need for bowel preparation and have lower morbidity and mortality rates but have high incidence of recurrence [35]. Where the decompression fails and there are signs of colonic gangrene, sigmoid resection and Hartmann’s procedure or double barreled colostomy is done to avoid the high mortality associated with primary anastomosis in this situation [35,36]. Recently laparoscopic resection has been used in high-risk or elderly patients who may not tolerate conventional surgery [36]. A more critical appraisal is however needed for its general use. The treatment of choice at this time is resection with primary anastomosis in patients with viable sigmoid colons and Hartmann’s procedure in those with gangrenous bowel [37]. Recurrence of sigmoid volvulus among patients treated with nonoperative approach is a common happening which ought to influence the choice of procedure to be performed. Most of our patients are poor and cannot afford to come back for interval sigmoid colectomy once the volvulus is reduced at sigmoidoscopy. It is for this reason that there is a tendency to perform resection and primary anastomosis. Irrespective of the presentation the major determining factor for primary resection and anastomosis is the presence or absence of complication such as gangrene or perforation. Many authors now prefer one stage primary resection and anastomosis procedure and colostomy if there are complications [10,16,18]. Colostomy is often advised in cases where the gut is gangrenous [13-16]. In the present study, the majority of patients (69.9%) were treated with resection and primary anastomosis, of which 63.0% were emergency cases. Colostomy was offered to 30.1% of cases who had gangrenous and perforated bowel. This treatment modality agrees with what was demonstrated by Okello *et al* [14] in Uganda. In this study, we found no significant difference between cases treated by primary anastomosis (one-stage resection) and those treated by two-staged resection (colostomy).This finding was consistent with that reported by Akcan *et al* [16] and Okello *et al* [14], that there is often no significant statistical difference whether a patient is treated with primary resection anastomosis or colostomy in terms of morbidity, complications and mortality. However, we found no prospective randomized studies done to compare the outcome in primary anastomosis and two-stage resections. Our study has confirmed that, in the hands of competent and experienced surgeons, resection and primary anastomosis in sigmoid volvulus patients with viable bowel, is safe and does not necessarily result in increased risk or a longer hospital stay. When the bowel is gangrenous, it is safer to resect and leave a temporary colostomy. In cases of ileo-sigmoid knotting, the secret behind a successful outcome lies in early diagnosis and prompt and appropriate treatment.

The presence of complications has an impact on the final outcome of patients presenting with bowel obstruction due to sigmoid volvulus. In keeping with other studies [11,14,23], surgical site infection was the most common postoperative complications in the present study. In our series, the complication rate was significantly higher in emergency operations than in elective operation and in patients with gangrenous bowel undergoing bowel resection.

The median duration of hospital stay in our study was 12 days, which is higher than that reported in other studies [14,23]. The length of hospital stay was significantly longer in patients with advanced age, concomitant medical illness and presence of complications. However, due to the poor socio-economic conditions in Tanzania, the duration of inpatient stay for our patients may be longer than expected.

Overall, the mortality of sigmoid volvulus in our setting was 17.1%, a figure that is slightly higher than 15.9% and 15.8% reported by Okello *et al* [14] and Oren *et al* [18]. Many other authors have reported mortality rates within the same range [10,11,23]. The high mortality rate in our study may be attributed to advanced age, presence of concomitant medical illness, late presentation (≥24 hours), presence of shock on admission and presence of gangrenous bowels. Addressing these factors responsible for high mortality in our patients is mandatory to be able to reduce mortality associated with this disease.

A total of 80.2% of our patients recovered well and were discharged. This figure is comparable with 84.1% reported by Okello *et al* [14] in Uganda. However, in this study the follow-up of patients was generally poor as more than half of patients (survivors) were lost to follow-up by the end of study period. Self discharge by patient against medical advice is a recognized problem in our setting. Similarly, poor follow up visits after discharge from hospitals remain a cause for concern. These issues are often the results of poverty, long distance from the hospitals and ignorance. Delayed presentation, discharge against medical advice and the large number of loss to follow up were the major limitations in this study. The fact that this study included only patients who were evaluated and treated at a single institution, findings from this study may not reflect the whole population in this region. Also, the prevalent of HIV infection (which was not assessed) in our setting is still high and this might have contributed to high mortality among our patients. However, despite this limitation, the study has provided local data that can help healthcare providers in the management of patients with sigmoid volvulus. The challenges identified in the management of sigmoid volvulus in our setting need to be addressed in order to deliver optimal care for these patients.

## Conclusion

Sigmoid volvulus remains the commonest cause of colonic bowel obstruction at Bugando Medical Centre and contributes significantly to high morbidity and mortality. Most of the patients presented acutely, requiring immediate resuscitation and surgical approach. It is suggested that in viable bowel, sigmoid resection and primary anastomosis is feasible as it may not adversely affect outcome. Temporary colostomy should be considered if the bowel is gangrenous or perforated. Early diagnosis and timely definitive treatment are essential in order to decrease the morbidity and mortality associated with this disease.
